# Genome Sequence of *Arthrobacter* Phage StuartMinion

**DOI:** 10.17912/micropub.biology.001950

**Published:** 2026-03-31

**Authors:** Moriah A. Adamson, Hailey M. Matsueda, Ava S.G. Shepp, Duy Linh Nguyen Tran, Noellani Shantel C. Aperocho, Trinity S. Baek-Kim, Ashley S. Dizon, Makala A. Gibson, Kalia M. Hendrickson, Yen Nhu K. Lu, Keira Mcnulty, Mia S. Molina, Elijah K. Neighbarger, Amber Redwine, Denise K. Takenaka, Kyli N. Taketa, Taylor A. Walker, Cherise Spotkaeff, Rin Wallace, Stuart Donachie, Alexander Culley, Megan L. Porter

**Affiliations:** 1 School of Life Sciences, University of Hawaiʻi at Mānoa, Honolulu, Hawaii, United States; 2 Pacific Biosciences Research Center, University of Hawaiʻi at Mānoa, Honolulu, Hawaii, United States; 3 Biological Electron Microscope Facility, University of Hawaiʻi at Mānoa, Honolulu, Hawaii, United States

## Abstract

Bacteriophage StuartMinion, isolated from an environmental sample collected in Kunia, Hawaiʻi, has a siphovirus morphology and is capable of infecting *Arthrobacter globiformis* B-2979. The genome is 35,207 bps, with 61 predicted genes and a GC content of 66.40%. Assigned to the AS3 subcluster of actinobacteriophages based on gene content, its genome is notably shorter than the average genome (38,373 bps) of other AS3 bacteriophages.

**Figure 1. Bacteriophage StuartMinion virion morphology:** Transmission electron micrograph of StuartMinion generated using negative staining (1% uranyl acetate) and transmission electron microscopy at the Biological Electron Microscope Facility, University of Hawaiʻi at Mānoa.

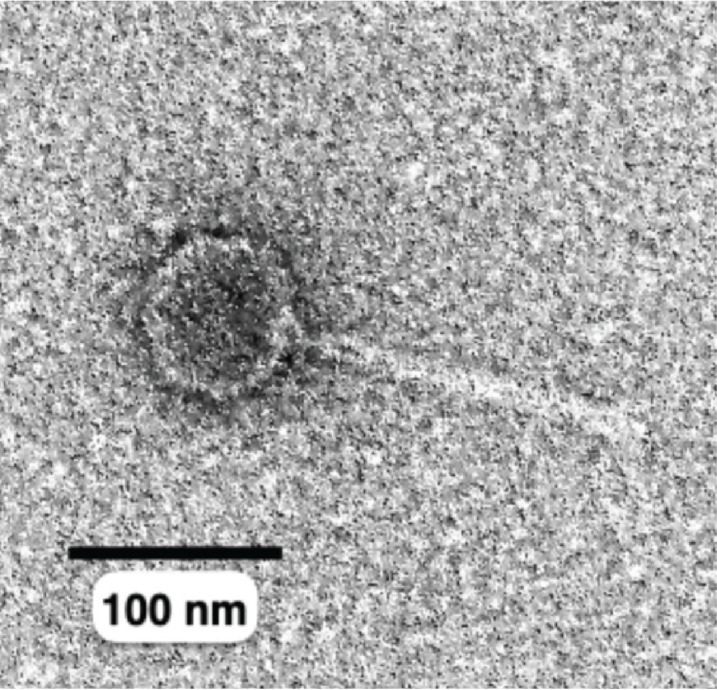



## Description

The isolation and sequencing of new bacteriophages leads to an increase in understanding of phage diversity and potential applications in areas such as agriculture, medicine, and biotechnology (Monk et al. 2010). Here we present the genome of bacteriophage StuartMinion isolated on *Arthrobacter globiformis* B-2979.

StuartMinion was isolated from a mud sample collected in Kunia, HI, USA (21.469321 N, 158.053371 W). The sample was suspended in PYCa medium, vortexed, centrifuged, and filtered (0.22 um pore size). The filtrate was inoculated with *A. globiformis* B-2979 and incubated with shaking at 30˚C for 72 hours. The culture was then filtered and the filtrate plated on LB top agar with *A. globiformis* B-2979. After incubation for 48 hrs at 30˚C, clear plaques 1.0 - 2.4 mm in diameter (n = 64; average ± standard deviation = 1.7 ± 0.4 mm) had formed. StuartMinion was subsequently purified through three rounds of individual plaque plating before high titer lysate was harvested. Transmission electron microscopy revealed a siphovirus morphology, with a 52.8 nm capsid and a 133 nm tail (n=1) (Fig. 1).

DNA was extracted from the high titer liquid lysate using the Promega Wizard DNA Clean-up kit and prepared for sequencing using the NEB Ultra II Library Kit (Zorawik et al., 2024). DNA was sequenced at the Pittsburgh Bacteriophage Institute on an Illumina NextSeq1000 resulting in 2.7 million 100 bp single-end reads. Raw reads were trimmed with cutadapt 4.7 (using the option: -nextseq-trim 30) and filtered with skewer 0.2.2 (using the options: -q 20 -Q 30 -n -l 50) prior to assembly in Newbler v2 (Roche) (Miller et al. 2010), and finished using Consed v29 (Gordon and Green, 2013; Russell 2017) resulting in a single genomic contig with 7350-fold coverage. The genome has a total length of 35,207 bps, a GC content of 66.4%, and a 12 base 3' single-stranded overhang (5'-GAGTTGCCGGCA).

Auto-annotation of the StuartMinion genome was completed using Glimmer v3.02b (Delcher et al., 2007) and GeneMarkS v2.5p (Besemer and Borodovsky, 2005). The Phage Evidence Collection and Annotation Network (PECAAN) v20241104 ( https://discover.kbrinsgd.org ) and Starterator v562 (http://phages.wustl.edu/starterator) were used to manually refine gene start sites. Phamerator Actino_draft database v560 (Cresawn et al., 2011) was used to review synteny. Potential functions for predicted protein coding genes were determined based on top hits from HHpred (PDB_mmCIF70, SCOPe70, Pfam-A, and NCBI_Conserved_Domains databases) (Zimmermann et al., 2018) and BLAST searches against the NCBI non-redundant protein and Actinobacteriophage protein databases (Altschul et al., 1990; Camacho et al., 2009; Russell and Hatfull, 2017). Finally, ARAGORN v1.2.38 (Laslett and Canback, 2004) was used to annotate tRNAs. Default parameters were used for all software.

StuartMinion was assigned to cluster AS, subcluster AS3, based on gene content similarity of at least 35% to phages in the Actinobacteriophage database, phagesdb ( https://phageDB.org ) (Pope et al., 2017; Russell and Hatfull, 2017). As with other AS3 phages, all predicted genes are transcribed unidirectionally with the exception of a central region of genes transcribed in the opposite direction. Within this central region, StuartMinion lacks ~ 3 kbp compared to other AS3 phages. As a result, StuartMinion has a much shorter genome containing only 61 predicted genes, as compared to the average 68.9 genes found in other AS3 bacteriophages, of which 39 were assigned putative functions. No tRNAs were identified. The 3 kbp absent from StuartMinion encode genes in other AS3 phages, including a predicted HNH endonuclease and a DNA binding protein. This region is transcriptionally downstream of predicted tyrosine integrase and immunity repressor genes which are conserved throughout cluster AS3. We therefore predict StuartMinion will likely be temperate similar to other AS3 phages (Jackson and Vega, 2025) despite this large deletion.

&nbsp;

**Nucleotide sequence accession numbers**

StuartMinion's genome sequence is available at GenBank (Accession No. PV876965 ) and the Sequence Read Archive ( SRX28943181 ).
